# Reemergence of Measles in the Americas: The Genotype B3 2011–2012 Outbreak in Ecuador

**DOI:** 10.3390/vaccines5020015

**Published:** 2017-06-02

**Authors:** Nicole K. Le, Rahul Mhaskar, Ismael Hoare, Mauricio Espinel, María Fernanda Rivadeneira, Sharad Malavade, Ricardo Izurieta

**Affiliations:** 1 Morsani College of Medicine, University of South Florida, Tampa, FL 33612, USA; 2 Center for Evidence Based Medicine and Health Outcomes Research, University of South Florida, Tampa, FL 33612, USA; rmhaskar@health.usf.edu; 3 Department. of Global Health, College of Public Health, University of South Florida, Tampa, FL 33612, USA; ihoare@health.usf.edu (I.H.); rizuriet@health.usf.edu (R.I.); 4 School of Medicine, Universidad Laica Eloy Alfaro de Manabi, Manta 13052732, Ecuador; espinel.mauricio@yahoo.com; 5 School of Medicine, Pontifical Catholic University of Ecuador, Quito 170109, Ecuador; maferivadeneira@yahoo.com; 6 Department of Internal Medicine, Brandon Regional Hospital, Brandon, FL 33511, USA; smalavad@health.usf.edu

**Keywords:** measles, vaccinations, Ecuador, indigenous

## Abstract

This study characterizes a measles outbreak which occurred in Ecuador in 2011–2012, analyzing data from 3700 suspected cases of measles reported to Ecuador’s Ministry of Public Health. The study population had a large age range and included 333 confirmed cases of measles. The greatest number of cases were found in the <1 year (32.43%, *n* = 108) and 1–4 year (30.03%, *n* = 100) age-groups. Compared to Mestizos, indigenous people had the highest number of cases (68.2%, *n* = 227), as well as a higher risk of infection (OR 7.278 (CI 5.251–10.087)). The greatest protection from measles was observed in individuals who received two doses of the measles vaccine. Residents of Pastaza (OR 6.645 CI (3.183–13.873)) and Tungurahua (OR 8.346 CI (5.570–12.507)) had a higher risk of infection than the other provinces. Of the 17 laboratory confirmed cases, all were identified as genotype B3. Age-group, ethnicity, measles vaccinations, and residence in Tungurahua and Pastaza were correlated with rates of measles infection in the outbreak. Tungurahua and Pastaza, where the outbreak originated, have large indigenous populations. Indigenous children <1 year of age showed the highest incidence. It is likely that indigenous women do not have immunity to the virus, and so are unable to confer measles resistance to their newborns.

## 1. Introduction

Of all the regions in the world, the Americas was the first to eliminate measles [1]. This interruption of the endemic transmission of measles was no easy task, considering that it is a highly contagious viral disease [2]. Following Cuba’s successful vaccination programs, a plan was enacted at the XXIV Pan American Sanitary Conference in 1994 with the goal of eliminating the measles virus (MeV) in the Americas by the year 2000 [3,4].

To achieve this, countries enacted the Pan American Health Organization (PAHO) three step strategy [3]. First, they started with a national “catch-up” vaccination campaign in order to rapidly increase immunity and interrupt the transmission of MeV. They did so by vaccinating all children aged 1–14 years regardless of disease and vaccination history [3]. The second step was to “keep-up” vaccinations. Routine vaccination services were strengthened to provide at least 95% coverage of all one-year-olds [3]. Since encounters with the virus were less likely after the “catch-up” campaign, the routine vaccination age was increased from nine months to one year of age, further increasing the efficacy of the vaccine [3]. In order to ensure high vaccination rates, quadrennial “follow-up” vaccination campaigns targeted children aged 1–5 years regardless of previous infection or vaccination history [3].

An additional strategy, “mop-up” vaccination campaigns, targeted groups of unvaccinated children, particularly in urban or hard-to-reach rural areas [3]. These campaigns also targeted regions with outbreaks of measles cases, weak surveillance of measles, low access to health services, and regions with high poverty and migration populations [5]. High risk groups having low coverage with the standard two-dose vaccination schedule included: impoverished regions with high population density and poor sanitation; communities philosophically opposed to immunization (e.g., indigenous populations which only trusted their traditional healers or shamans, Amish communities, or the anti-vaccine movement); young migratory adults who travel from low density rural areas to highly dense urban areas; persons working or living in close proximity to tourist destinations and in the tourist industry; geographically remote populations; college students; medical professionals; and international travelers [2,6,7,8,9,10]. It was found that house-to-house vaccination, along with house-to-house surveillance of measles, were the most efficient approaches for reaching high vaccination rates in at-risk populations [11].

To increase coverage, immunization programs provided vaccinations to entire households [7]. In the 2000s, efforts to promote the elimination of MeV were strengthened by the joint implementation of measles and rubella ‘‘speed-up’’ campaigns that also targeted women of childbearing age (12–29 years) [12]. The objectives were to reduce the risk for congenital rubella syndrome and ensure mothers had immunity that could be conferred to their infant during the first 6–12 months of life [13]. Through this strategy, the aim was to transfer maternal antibodies to protect the infant until the vaccination scheduled on the first birthday.

Considering the current progress toward the elimination of MeV, the most pressing concern is to sustain those achievements which requires governments to maintain and raise coverage rates through national immunization programs [1]. This current progress may be increased through effective surveillance and further mass immunization programs [1]. To maintain reliable herd immunity from measles, immunization coverage rates need to be at least 90% [12]. Obstacles to the elimination of MeV include the accumulation of susceptible children over time and the continual circulation of the MeV in other regions of the world, particularly in Southeast Asia, Africa, and Europe [7,12]. Notably, all genotypes, from the outbreaks since 2003, have been identified as nonindigenous to the Americas [14,15].

The 2011–2012 outbreak in Ecuador was quite alarming, as the country had been MeV free since 1997 [16]. This MeV free period was made possible by large vaccination efforts and was supported by the implementation of the National Vaccine Law which was enacted in 1999. This particular law mandated funding for nationwide vaccination campaigns, including both the administration of existing vaccines and newly developed vaccines [8]. Through this law, the Organic Health Law, and Ecuador’s Constitution, vaccinations were guaranteed as a public good, ensuring legal support for the Expanded Program on Immunization [12].

The study describes the key characteristics of the measles outbreak which occurred in Ecuador during 2011–2012. It outlines the risks to the Mestizo and Indigenous populations in Ecuador.

## 2. Materials and Methods

The information for this research was obtained from the database of the National Surveillance System Eruptive Non Vesicular Disease (EFENV), the Ministerio de Salud Publica (MSP) of Ecuador (Ministry of Public Health). This retrospective cross sectional outbreak study was approved by the Institutional review board (no. Pro00029260) at the University of South Florida. The data from the 2011–2012 measles epidemic in Ecuador was collected by the MSP of Ecuador under the Expanded Immunization Program. This included clinical characteristics of cases and suspected cases of measles. A case of measles was defined as a laboratory confirmed case of measles, or a primary contact of a laboratory confirmed case of measles exhibiting signs and symptoms of measles. Seventeen suspected cases of measles were tested using laboratory tests (blood, nasopharyngeal swab, and urine tests) to confirm infection with MeV, and genotyping. All the samples were processed in the MSP central laboratories. Information was collected on the vaccination status of the individual, number of doses of MeV vaccine received, age group, gender, province of residence, and ethnicity (see the Supplementary Materials file).

The study population included 3700 individuals reported as suspected cases of measles to the measles surveillance system. The inclusion criteria was a classification as “suspicious” by the EFENV for measles, presenting with exanthema and fever, between the years of 2011 and 2012. The exclusion criteria was the absence of the classical signs of measles (i.e., fever and rash), as reported by the surveillance system EFENV. The sampling frame consisted of patients with suspected measles, which were detected and reported by health institutions, public or private, and entered into the EFENV Surveillance System from 2011 until 2012. The statistical software SPSS v22 and SAS v9.4 were used, accessed from the University of South Florida. Descriptive statistics using frequency counts and Chi-square analyses were done to assess the study sample characteristics. Logistic regression analyses using stepwise selection was performed to identify factors associated with measles infection.

## 3. Results

**Figure 1:**
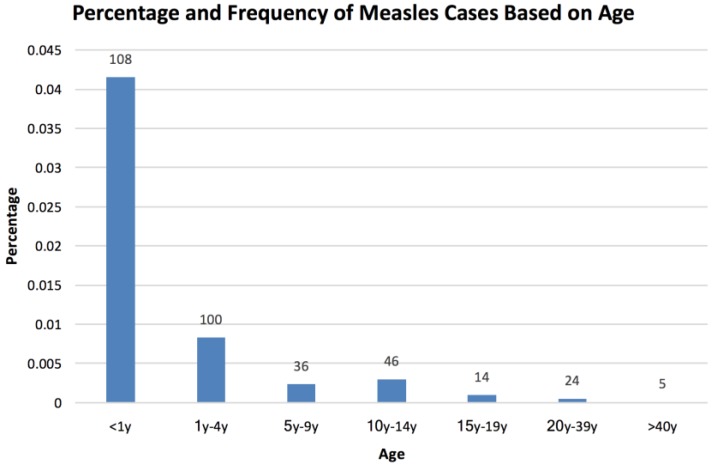
Percentage and frequency of measles cases based on age. Out of the 333 measles cases, the percentages and number of people within each age group are presented. The percentages have been adjusted for each age group over the entire Ecuadorian population. The frequency of each group is labelled above each bar.

Of the 3700 patients, 333 patients met the case definition by laboratory confirmation or epidemiologic linkage. Of the 333 cases, 49.5% were males and 50.5% were females. The ethnicities of the measles cases were 68.2% Indigenous (Amerindian descendants) and 31.8% were Mestizos (mixed race of Spanish and Amerindian descendants). When comparing the percentage of the measles cases within the entire population of Ecuador, patients <1 year comprised of 0.0415%, patients 1 year to 4 years involved 0.0083%, patients 5 years to 9 years included 0.0024%, patients 10 years to 14 years consisted of 0.0030%, patients 15 years to 19 years made up 0.0010%, patients 20 years to 39 years were 0.0005%, and patients 40 years and older were 0.0001% of the population (Figure 1).

**Figure 2:**
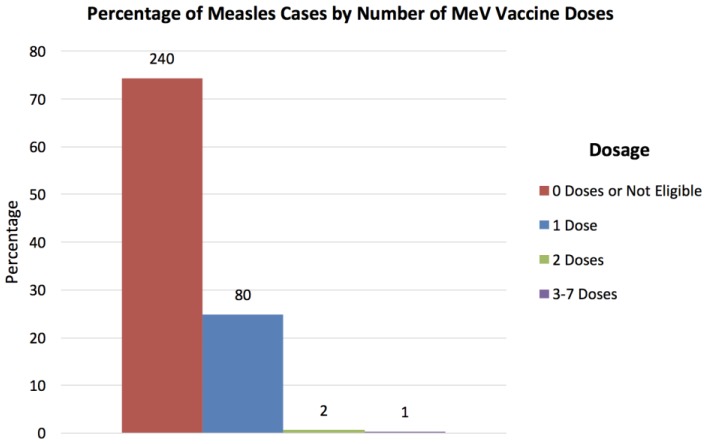
Percentage of measles cases by number of MeV vaccine doses. Out of the 333 measles cases, the percentages and number of people are presented for each number of MeV vaccine dose. The frequency of each group is labelled above each bar. People not eligible for the vaccine were grouped with “0 Doses”.

**Figure 3:**
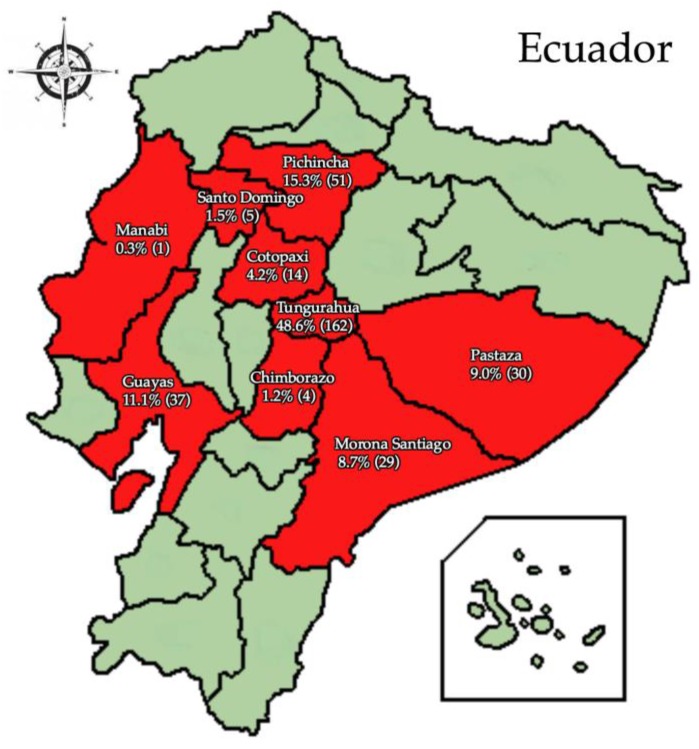
Percentage of measles cases by province in Ecuador. Out of the 333 measles cases, the percentages of cases each province had is shown. The frequency of cases in each province is also included in the parentheses.

Of the people with measles cases, 74.3% had zero doses of the MeV vaccine, 24.8% only had 1 dose, 0.6% had 2 doses, and 0.3% had 3–7 doses. Of the 74.3% who did not receive any vaccine dose, 60.1% did not provide a reason while 14.2% were not eligible for the vaccine as they were younger than 12 months, pregnant, or had a concomitant disease. (Figure 2). Figure 3 shows the percentage of measles cases in each province. Of the nine provinces that had cases of measles, 48.6% were residents of Tungurahua, 15.3% from Pichincha, 11.1% from Guayas, and 9.0% from Pastaza. Morona Santiago, Cotopaxi, Santo Domingo, Chimborazo, and Manabi had the remaining cases of measles.

**Figure 4:**
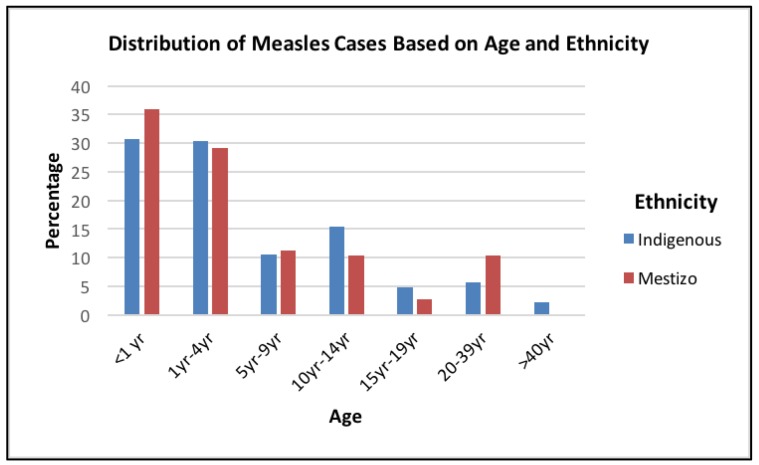
Distribution of measles cases based on age and ethnicity. Out of the 333 measles cases, the percentage of both Indigenous and Mestizo persons are shown within each age group.

Of the people with measles, 49.7% of the Mestizos and 57.9% Indigenous persons had never received a MeV vaccine (Table 1). The genotype B3 was found for all 17 laboratory-confirmed cases. Figure 4 demonstrates that 30.8% of the indigenous and 35.9% of the Mestizos with measles were younger than one year. The next highest incidence of measles was within the 1–4 years age group with 30.4% being indigenous and 29.2% were Mestizos (Figure 4).

When determining the risk of getting measles, people with no vaccine had an odds ratio and 95% confidence interval of 34.85 (8.001, 151.62) when compared to those with two doses of the vaccine. The odds ratio of indigenous compared to Mestizos was 7.28 (5.25, 10.09). Out of the provinces, when compared to Azuay, the provinces with the highest odds ratio were Pastaza with an odds ratio of 6.65 (3.18, 13.87) and Tungurahua with an odds ratio of 8.35 (5.57, 12.51).

## 4. Discussion

This study found that the greatest risk factor for contracting measles was lack of vaccination. In addition, the indigenous persons in Ecuador had a greater risk of contracting measles. Factors which may have contributed to this increased risk include: poor access to preventive services, living in remote geographic locations which can impede outreach program efforts, the difficulty of maintaining vaccine refrigeration chains, poverty, cultural differences, and malnutrition [17]. The provinces at highest risk, including Pastaza and Tungurahua, have large indigenous populations. Mestizos have, on average, a higher socio-economic status compared to the indigenous populations in Ecuador [18]. These inequities, especially in the health system, greatly impact the indigenous populations and may exacerbate a measles outbreak [17].

Children under the age of five were at the greatest risk for measles. Children under one year with measles lacked maternal immunity to the virus because their mothers were never vaccinated nor exposed to the MeV. The vaccination strategy for Ecuador for the year 2010 mandated two doses total at age one year and six years [19]. With a lack of maternal immunity, children under one year were vulnerable until their first dose of the vaccine [13,19]. Due to the increased rates of measles infection within the 1–4 years group, it is likely that these children also did not receive the MeV vaccine.

Measles did not affect one gender more than the other. We found that the number of doses received was negatively correlated with the risk of infection. There was a slight increase in measles cases for people who received 3–7 doses of the vaccine. A possible explanation for this was poor record keeping, as the standard dosing schedule is two doses total.

When the MeV were genotyped, the B3 genotype was found, which is endemic to Africa and parts of Europe. Individuals from Europe, particularly Spain, and Africa, may have imported the virus into Ecuador. Large populations of Ecuadoreans live in Spain, as a result of the mass emigration from Ecuador that occurred during the economic crisis of 1990 [20]. Ecuadoreans returning from Spain may have been infected with the virus during an outbreak and brought it to Ecuador. The government may have classified many of these Ecuadoreans as tourists because they applied for tourist visas for their visits, rather than filing as returning Ecuadorian citizens due to the former method’s relative simplicity of entry. A large portion of the people who migrated were the indigenous population seeking employment in domestic service, construction, and agriculture in Spain [21]. It is likely that a good number of them were not vaccinated prior to their emigration, and the low vaccination coverage rates in Europe left them vulnerable to MeV [22].

Furthermore, African migrants started migrating to Ecuador when the Ecuadorean government lifted all travel visa requirements in 2008 [23]. The influx of Africans, who may have been carrying the MeV, may have contributed to the outbreak. Most of the outbreaks in the Americas have been linked to the large number of outbreaks affecting Africa and Europe [24]. The long period of absence of measles cases ended in the 26th epidemiological week of 2011 [25]. The MeV genotype B3’s presence, seen circulating in Spain and Kenya, was confirmed in Ecuador [26,27].

The South African FIFA World Cup in June–July 2010 was a possible explanation for the presence of MeV genotype B3 virus in Ecuador, since it is a commonly held theory in epidemiology that transmission of contagious diseases increases during major international gatherings, such as the World Cup and other similar events [12,28]. However, the idea that the World Cup led to the outbreak is unlikely due to the gap in time between the World Cup and the measles outbreak. The MeV incubation period is only a mere 10–12 days, not an entire year [2].

To prevent future outbreaks, increased vaccination coverage, especially among the indigenous population and younger children, should be increased. Vaccination of females in the reproductive age group would ensure that maternal immunity is passed on to her child so that children younger than one year would be better protected, and herd immunity is further strengthened. In addition, epidemiological surveillance systems need to be strengthened to better respond to future outbreaks.

Finally, we advise against a reduction of surveillance and vaccination activities that are necessary for the elimination of MeV. Often, when a disease is eliminated from a country or region, the governments stop viewing the disease as a real and urgent threat [29]. As a result, disease monitoring and vaccination programs are abandoned, laxly enforced, or underfunded. This leaves countries, especially developing nations, vulnerable to the reintroduction of MeV. We advise that, even after elimination in a particular region, governments should remain vigilant in enforcing their vaccination policies, as the virus is still circulating in other parts of the world.

When surveillance and vaccination efforts are maintained for long periods of time, we can eliminate the virus from progressively larger areas. After numerous spikes in measles outbreaks across the Americas, countries on both continents have collaborated to interrupt endemic transmission of MeV. They increased vaccination efforts and surveillance, and in September 2016, the Pan American Health Organization declared that the region of the Americas had eliminated MeV [30].

Our study was limited by missing data points for several of our variables. To improve, a more complete data set would be preferable for more robust conclusions. Furthermore, while there were 333 confirmed cases of measles, only 17 of these cases were lab confirmed. Even though the remaining 316 cases were primary contacts of the lab confirmed cases with the classic signs and symptoms of measles, it is possible that they either were not cases of measles, or not the B3 genotype.

## 5. Conclusions

The reemergence of MeV was unexpected in the Americas, especially in Ecuador, after previously achieving elimination. The B3 genotype was found to be predominant during the 2011–2012 outbreak, and the greatest risk factor for its reemergence was the lack of vaccination. The people most likely to contract MeV during the outbreak were children five years and younger, while the indigenous populations were found to be more at risk than Mestizos. Despite much progress, more work is needed before the elimination of MeV becomes a reality. The Americas have set a good example to follow, and with the necessary amount of international cooperation and effort, the global elimination of MeV may soon be achieved.

## Figures and Tables

**Table 1:** Distribution of study population based on ethnicity and vaccination status. Out of the entire study population, the percentages of Indigenous and Mestizo persons with and without vaccinations are shown. The frequency of each group is labelled above each bar.

Vaccine Status	Indigenous		Mestizo	
	Percentage	Frequency	Percentage	Frequency
**Did not receive measles vaccine**	57.85%	280	49.67%	1375
**Received measles vaccine**	42.15%	204	50.34%	1393

## Supplementary Materials

The following are available online at www.mdpi.com/2076-393X/5/2/15/s1.
